# A systematic review of clinical outcomes for outpatient vs. inpatient shoulder arthroplasty

**DOI:** 10.1177/17585732211007443

**Published:** 2021-04-28

**Authors:** Edward Perera, Breanne Flood, Kim Madden, Danny P Goel, Timothy Leroux, Moin Khan

**Affiliations:** 1 Epsom & St. Helier University NHS Hospital, London, UK; 2 Research Institute of St. Joseph’s Healthcare Hamilton, Hamilton, Canada; 3 Division of Orthopaedic Surgery, Department of Surgery, McMaster University, Hamilton, Canada; 4 Department of Orthopedic Surgery, University of British Columbia, Vancouver, Canada; 5 Division of Orthopaedic Surgery, University of Toronto, Toronto, Canada

**Keywords:** Outpatient, shoulder arthroplasty, adverse events, clinical outcomes, cost analysis

## Abstract

**Background:**

Outpatient shoulder arthroplasty is growing in popularity as a cost-effective and potentially equally safe alternative to inpatient arthroplasty. The aim of this study was to investigate literature relating to outpatient shoulder arthroplasty, looking at clinical outcomes, complications, readmission, and cost compared to inpatient arthroplasty.

**Methods:**

We conducted a systematic review of Medline, Embase and Cochrane Library databases from inception to 6 April 2020. Methodological quality was assessed using MINORS and GRADE criteria.

**Results:**

We included 17 studies, with 11 included in meta-analyses and 6 in narrative review. A meta-analysis of hospital readmissions demonstrated no statistically significant difference between outpatient and inpatient cohorts (OR = 0.89, *p* = 0.49). Pooled post-operative complications identified decreased complications in those undergoing outpatient surgery (OR = 0.70, *p* = 0.02). Considerable cost saving of between $3614 and $53,202 (19.7–69.9%) per patient were present in the outpatient setting. Overall study quality was low and presented a serious risk of bias.

**Discussion:**

Shoulder arthroplasty in the outpatient setting appears to be as safe as shoulder arthroplasty in the inpatient setting, with a significant reduction in cost. However, this is based on low quality evidence and high risk of bias suggests further research is needed to substantiate these findings.

## Introduction

Shoulder arthroplasty can provide significant improvement in quality of life for patients with end stage glenohumeral joint disease.^1^ The number of shoulder arthroplasties performed annually are increasing given an ageing population, expanded indications, improved techniques and advances in implant design.^2^ Given this rise, demand on health services is rising and likely to put an increased strain on hospital resources and expenditure in the upcoming years.^3^ In response to this and increased patient interest, outpatient shoulder arthroplasty is growing in popularity.^4^ Previous barriers to performing arthroplasty in the outpatient setting were concerns over the management of pain, bleeding and post-operative medical complications; however, improvements in pre-operative optimisation of patients, perioperative management of pain with use of anaesthetic blocks,^5^ and overall improvements in surgical technique^6,7^ and intraoperative management of blood loss^8^ have addressed some of these concerns. Despite this, there are those who express concerns that outpatient shoulder arthroplasty may result in increased complications, and ultimately may be more costly due to an increase in readmissions.^9^ Outpatient shoulder arthroplasty has the potential to reduce expenditure and resource allocation on appropriately selected patients; however, it must pose no increased risk of post-operative adverse events when compared to inpatient shoulder arthroplasty. To date, no review has taken a systematic approach to review data regarding outpatient shoulder arthroplasty. We aim to compare outcomes, complications, Emergency Department (ED) presentations, readmissions, and cost of outpatient and inpatient shoulder arthroplasty.

## Materials and methods

The study and protocol were registered with PROSPERO (Prospero ID: CRD42020183201) and followed the reporting guidelines in the Preferred Reporting Items for Systematic Reviews and Meta-Analyses Protocols (PRISMA-P) statement.^10^

### Search strategy

We conducted a comprehensive search of electronic databases (Medline, Embase and Cochrane Library) from database inception to 6 April 2020. We used the following research terms: total shoulder arthroplasty, reverse shoulder arthroplasty and outpatient. Free text was supplemented with MeSH and Emtree terms to increase search sensitivity (Supplemental Table 1). Following removal of duplicates, two reviewers (EP and BF) independently screened articles for eligibility using title, abstract and full text. Screening was in duplicate, at the title and abstract level, with inclusion to be decided on the basis of at least one reviewer choosing to include. At the full text level any disagreement over inclusion was resolved by consensus discussion between reviewers, if no agreement could be made a third reviewer (KM) made the final decision.

### Inclusion and exclusion criteria

Inclusion criteria: (1) randomised controlled trials (RCTs) or observational studies; (2) adults who have undergone an anatomic total shoulder arthroplasty (aTSA), hemiarthroplasty (HA) or reverse total shoulder arthroplasty (rTSA); (3) articles published in peer-reviewed English language scientific journals; (4) procedures performed in the outpatient setting. Exclusion criteria included: (1) revision arthroplasty; (2) arthroplasty due to fracture.

### Data extraction

We extracted data in duplicate by two reviewers (EP and BF) using a data extraction form (Microsoft Excel, version 15.2, Microsoft Corporation, Redmond, WA, USA). We extracted the following data: journal, authors, year of publication, location of study, study design, level of evidence, statistical test used, sample size, gender, mean age of patients, BMI, comorbidities, Charlson Comorbidity Index (CCI)^11^ score, American Society of Anesthesiologists (ASA)^12^ grade, surgical indication, procedure details (incision type, blood loss, prosthesis used), perioperative analgesia and antibiotics given, outcomes measured including functional scores (ASES – American Shoulder and Elbow Surgeons),^13^ single assessment numerical evaluation (SANE),^14^ Visual Analogue Scale (VAS), range of motion (ROM), patient important outcomes, complications reported, post-operative healthcare (including ED presentations and readmissions), revisions of primary procedure, length of stay, total cost of procedure and admission, duration of follow-up, and any comparable control data available.

### Assessment of methodological quality

Risk of bias was assessed in duplicate using the Methodological Index for Non-Randomised Studies (MINORS) score, in which a maximum score for a comparative or non-comparative study is 24 out of 24, or 16 out of 16 respectively, suggesting the lowest risk of bias.^15^

### Statistical analysis

We used descriptive statistics (median, mean, standard deviation (SD) and percentages as appropriate) to summarise study characteristics. To assess inter-observer agreement we calculated a Cohen’s kappa (*κ*) coefficient at the full text stage according to guidelines laid out in Landis and Koch.^16^ We conducted formal random-effects meta-analyses using RevMan version 5.3, presenting forest plots with *I*^2^ heterogeneity statistics and pooled effects with 95% confidence intervals (CIs). We reported binary outcomes as pooled odds ratio (OR) with 95% CI. We present medical and surgical complications separately as we wanted to identify any variations from the standard post-operative course that were a direct result of surgical intervention. We constructed a GRADE summary of findings tables for all outcomes using GRADEPro GDT online software (Table 1).

**Table 1.** Grade summary of findings – outpatient procedures compared to inpatient procedures for shoulder arthroplasty.

Certainty assessment						No. of patients		Effect		Importance
No. of studies	Study design	Risk of bias	Inconsistency	Indirectness	Imprecision	Outpatient procedures	Inpatient procedures	Relative (95% CI)	Absolute (95% CI)	
Readmissions										
8	Observational studies	Serious^a^	Not serious	Not serious	Not serious	162/2435 (6.7%)	3305/55604 (5.9%)	OR 0.89 (0.63 to 1.25)	6 fewer per 1000 (from 21 fewer to 14 more)	Important
Revision										
7	Observational studies	Serious^a^	Not serious	Not serious	Not serious	40/2611 (1.5%)	406/27612 (1.5%)	OR 1.00 (0.72 to 1.39)	0 fewer per 1000 (from 4 fewer to 6 more)	Critical
Post-operative complications										
8	Observational studies	Serious^b^	Not serious	Not serious	Not serious	323/1729 (18.7%)	2963/18134 (16.3%)	OR 0.70 (0.52 to 0.94)	43 fewer per 1000 (from 71 fewer to 8 fewer)	Critical
Post-operative medical complications										
8	Observational studies	Serious^b^	Not serious	Not serious	Not serious	222/1729 (12.8%)	2062/18134 (11.4%)	OR 0.86 (0.74 to 1.01)	14 fewer per 1000 (from 27 fewer to 1 more)	Important
Post-operative surgical complication										
8	Observational studies	Serious^b^	Not serious	Not serious	Not serious	101/1729 (5.8%)	961/18134 (5.3%)	OR 0.71 (0.45 to 1.12)	15 fewer per 1000 (from 28 fewer to 6 more)	Critical

CI: confidence interval; OR: odds ratio.

a Bean et al. and Brolin et al. demonstrate increased risk of bias for lower scores in MINORS criteria.

b Bean et al., Brolin et al. and Nelson et al. demonstrate increased risk of bias for lower scores in MINORS criteria.

**Table 2.** Cost analysis.

Author, Year	Total cost of admission per patient OP (SD)	Total cost of admission per patient IP (SD)
Cancienne et al., 2017	30 days post-operative $14,722 (2806)	30 days post-operative $18,336 (3082)
Gregory et al., 2019	$22,907 (13,599)	$76,109 (48,981) Charges removed (incl nursing, medication, accommodation) $32,330 (24,221)
Ode et al., 2020	Median $37,395 HOPD $55,990 ASC $31,790	Median $62,905
Walters et al., 2019	$28,382.33	

ASC: ambulatory surgical centre; HOPD: hospital outpatient department; OP: outpatient; IP: inpatient; SD: standard deviation.

## Results

### Eligibility

**Figure 1.**
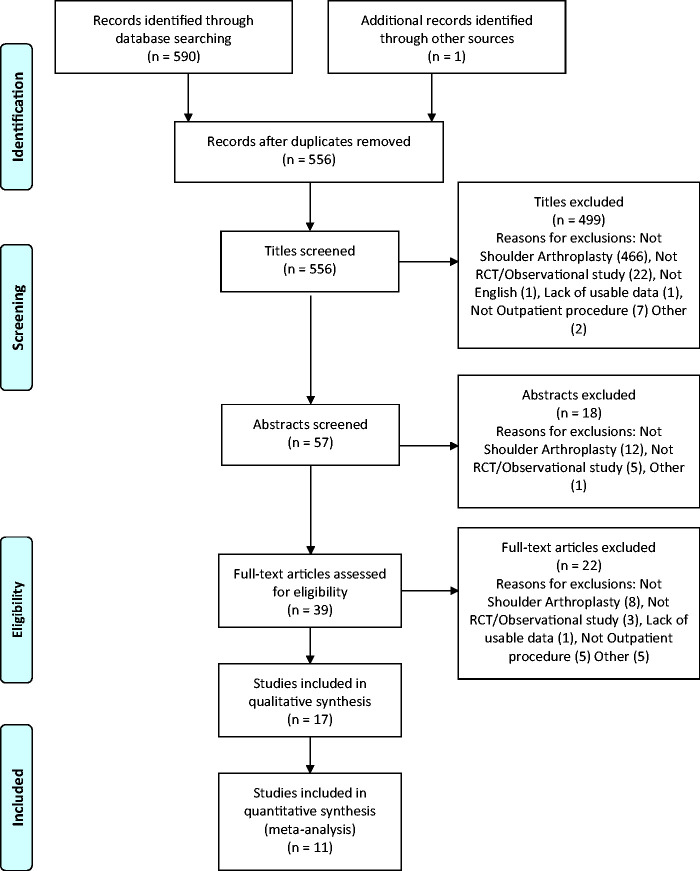
Flow diagram of study selection and exclusion.

We identified a total of 590 studies from a comprehensive search of databases. After removal of duplicates, 555 studies were suitable for screening (Figure 1). From this selection we included 17 studies in the final systematic review, of which 11 studies were used in the quantitative meta-analyses. Of the remaining six, there was insufficient overlap in reported outcomes to perform quantitative analysis, therefore these were discussed narratively.

Inter-observer agreement was substantial at full text screening with *κ* = 0.79 (CI: 0.60–0.98).

### Study characteristics & patient demographics

We included 17 studies (*n* = 98,980) in this review, of these 7 were database review studies with the remaining 10 studies having clinical cohorts. Six thousand eighteen of the included patients were from outpatient cohorts and 92,962 were from inpatient cohorts. All articles published reported on research performed in North America, with 16 studies originating in the United States and 1 study originating from Canada (Supplemental Table 2). All studies were non-randomised, with 15 out of the total 17 having a control group. Of the 17 studies, 8 studies reported on outcomes for an aTSA cohort; 1 study reported on outcomes for a rTSA cohort; 5 for outcomes of both aTSA and rTSA cohorts; 2 studies reported outcomes of aTSA, rTSA and HA cohorts combined; and 1 study excluded HA but did not specify their cohorts further. Of reported data, the overall mean age was 69.4 (7348 patients). Outpatient cohort patients were a mean age of 65.5 (1068 patients) whereas inpatient cohorts were 70.1 (6280 participants; Supplemental Table 2). Of all, 42.4% of participants (98,964) were male, which included 49.8% of outpatients (6010) and 41.9% of inpatient (92,954) being male. Mean BMI reported was 30.9 (1030), with a mean BMI of 30.4 in outpatients (544) and 31.4 in inpatients (486). The mean CCI score reported was 3.36 (22,937), with a mean CCI of 3.16 for outpatients (2446) and 3.38 for inpatients (20,491). Mean ASA grade reported was 2.36 (485), with a mean ASA of 2.25 for outpatient (305) and 2.54 for inpatients (180). Mean length of follow-up for studies was 194 days and ranged from seven days to two years.

### Study variability

Of the seven studies reporting operative technique all utilised a deltopectoral approach. Four of seven stated a subscapularis tenotomy was performed, with one of the seven studies performed both subscapularis tenotomy and biceps tenodesis. Of the total of 37,652 reporting implant used: 34,939 (92.8%) participants underwent aTSA, 2705 (7.2%) underwent rTSA and 8 (0.02%) had HA.^1,17–27^ Of all, 52.9% reported the use of nerve blocks as adjuncts to anaesthesia, this equated to 9.9% of all participants included having a documented nerve block (Supplemental Table 3). The top three most prescribed analgesics were oxycodone (38.9%), paracetamol/acetaminophen (33.3%) and gabapentin (27.8%). Of the 17 studies, 9 commented on post-operative care, with the remainder making no comment.^1,17,18,20,22,26–28^ The majority of these followed a similar pattern of advice with passive ROM exercises in a sling up to 6 weeks post-operatively; followed by gentle activities or active ROM between 6 and 12 weeks; and unrestricted activities 12 weeks post-operatively.^1,18,20,22,26–28^ Four studies also incorporated follow-up calls with participants to assess pain at either post-operative day one or three.^20,22,26,28^

### Adverse events

**Figure 2.**
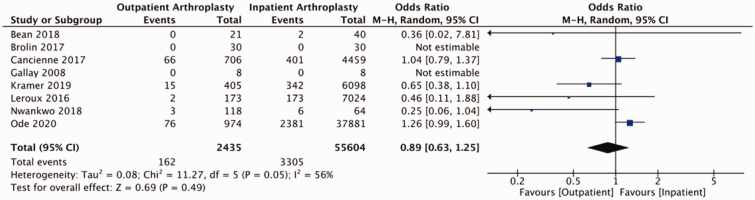
Forest plot of odds of readmissions comparing outpatient to inpatient shoulder arthroplasty.

**Figure 3.**
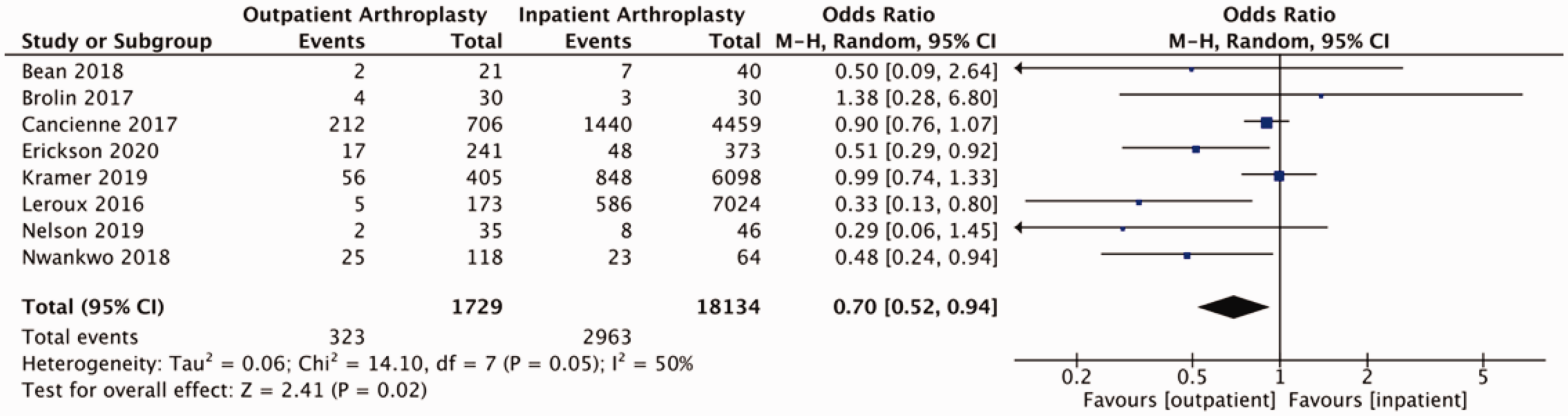
Forest plot for odds of any post-operative complications comparing outpatient to inpatient shoulder arthroplasty.

**Figure 4.**
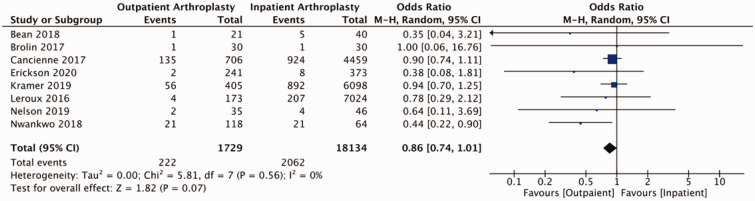
Forest plot for odds of medical complications comparing outpatient to inpatient shoulder arthroplasty.

**Figure 5.**
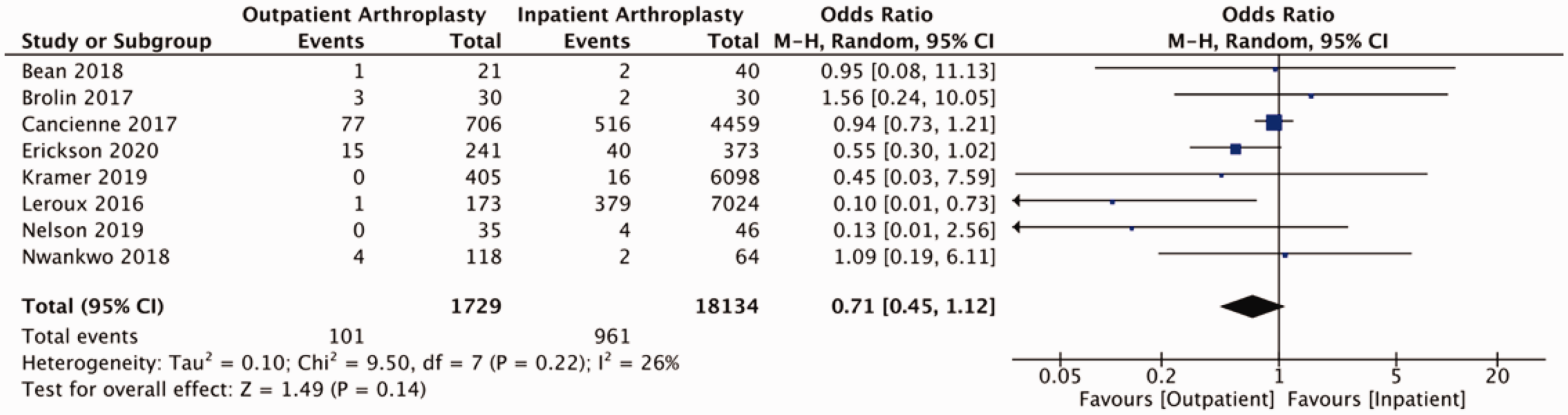
Forest plot for odds of surgical complications comparing outpatient to inpatient shoulder arthroplasty.

**Figure 6.**
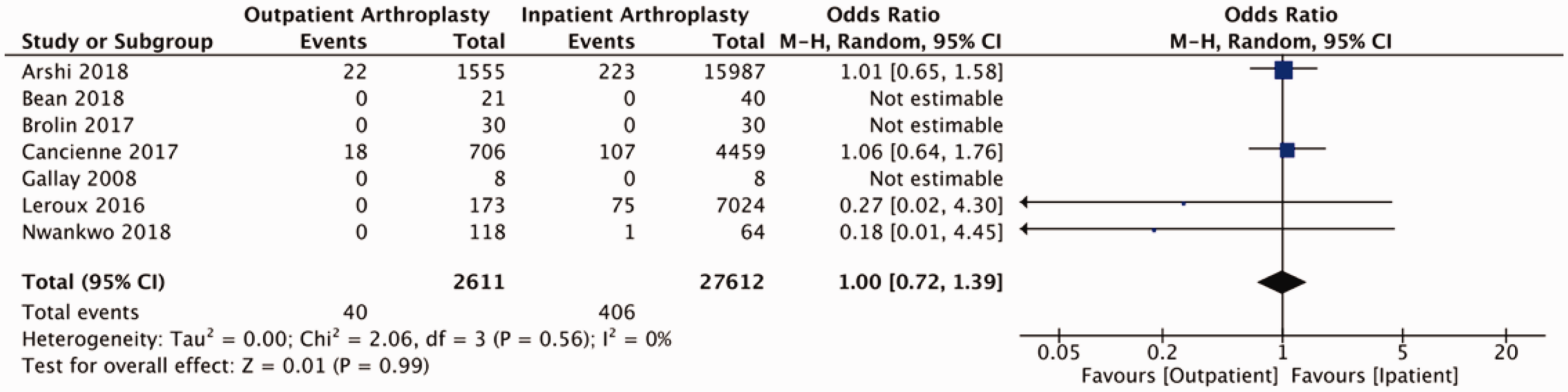
Forest plot of odds of revision of procedure comparing outpatient to inpatient shoulder arthroplasty.

Of studies reviewed, the majority reported on adverse events relating to shoulder arthroplasty in the form of readmissions, revisions, and medical and surgical complications (Supplemental Table 3). Pooled outcomes from eight studies (58 039 patients) did not demonstrate a statistically significant difference in readmissions to hospital between outpatient and inpatient cohorts (OR = 0.89, 95% CI: 0.63–1.25, *p* = 0.49, *I*^2 ^= 56%; Figure 2). Two studies reported on ED visits post-operatively. Kramer et al. reported 50 (12.3%) post-operative visits to ED in their outpatient cohort (405 patients) and 760 (12.4%) post-operative visits to ED in their inpatient cohort (6098 patients).^23^ Nwankwo et al. reported 19 (16.1%) post-operative ED visits in their outpatient cohort (118 patients) and 18 (28.1%) post-operative ED visits in their inpatient cohort (64 patients).^27^ Our analysis of post-operative complications (eight studies including 19 863 participants) demonstrated a statistically significant reduction in the odds of a complications for patients undergoing outpatient shoulder arthroplasty (OR = 0.70, 95% CI: 0.52–0.94, *p* = 0.02, *I*^2 ^= 50%; Figure 3). Separating for medical and surgical complications, pooled findings from eight studies (19 863 participants) did not detect a statistically significant difference in medical complication between outpatient and inpatient cohorts (OR = 0.86, 95% CI: 0.74–1.01, *p* = 0.07, *I*^2 ^= 0%; Figure 4) and pooled results from eight studies (19 863 patients) did not identify a difference with respect to surgical complications between groups (OR = 0.71, 95% CI: 0.45–1.12, *p* = 0.14, *I*^2 ^= 26%; Figure 5). Pooled findings from seven studies (30,223 patients) did not detect a statistically significant difference in the odds of requiring revision surgery (OR = 1.00, 95% CI: 0.72–1.39, *p* = 0.99, *I*^2 ^= 0; Figure 6).

### Clinical outcomes

Six of the 17 studies assessed patient clinical outcomes with respect to ASES score, VAS score, SANE score, ROM and patient satisfaction (Supplemental Table 4). Only one article compared both outpatient and inpatient cohorts with regards to pre- and post-operative ASES scores.^18^ Despite lower final ASES score, the inpatient cohort demonstrated greater mean improvement in ASES scores measured at both one- and two-years post procedure. The best reported outcomes were pre- and post-operative VAS scores, with four studies reporting on changes, of which three were comparative. All studies demonstrated a reduction in VAS scores following surgery, whilst Bean et al. suggested a greater improvement in scores for outpatient participants, the results from Erickson et al. and Gallay et al. were inconclusive and showed no difference between cohorts.^17,19,21^ Two studies^10,11^ reviewed post-operative SANE scores, one study^19^ compared outpatient and inpatient participants without demonstrating any clear benefit between cohorts. Two studies investigated post-operative ROM, but only in outpatient cohorts.^10,13^ Patient important outcomes were reviewed in three studies, where patients were asked to rate their satisfaction with their procedures, all suggested they were either satisfied or very satisfied, regardless of location.^13,17,18^ Cost analyses were conducted in four studies, with three involving a comparator group.^1,2,21,29^ All three comparison studies demonstrated a cost saving by performing procedures in the outpatient setting with costs ranging from 19.7% to 69.9% less per patient in the outpatient setting (Table 2). Gregory et al. demonstrated the highest saving per patient case, demonstrating a mean saving per patient of $53,202 (69.9%, *p* < 0.001) when performed in the outpatient setting.^30^ When removing inpatient specific charges, such as accommodation, nursing charges and medication, they demonstrated outpatient arthroplasty to offer a saving of $9423 (29.1%, *p* < 0.0001) per patient.

### Risk of bias

We assessed risk of bias using the MINORS criteria for all studies, none achieved a score of 24/24 (or 16/16 for non-comparative studies; Supplemental Table 2). The overall certainty of evidence according to GRADE criteria was very low, due to meta-analyses only containing observational studies and serious risk of bias.

## Discussion

The primary finding of this review is that in appropriate selection of patients based on age, health status, outpatient shoulder arthroplasty is as safe as arthroplasty performed in the inpatient setting. In addition, this review did not detect a statistically significant difference in readmissions and complications between outpatient and inpatient shoulder arthroplasty, but there was a significant cost-reduction associated with outpatient procedures. Despite the findings of these studies showing potentially no difference or perhaps benefits to outpatient shoulder arthroplasty, these findings are based on low-quality evidence and the majority of available literature being observational data.

With a trend towards increasing outpatient procedures over time, it is of utmost importance not to compromise patient safety. Brolin et al. were the first to suggest that outpatient shoulder arthroplasty presented no increased risk of post-operative complications when compared to inpatient procedures.^28^ Of the 17 studies, 4 studies did not collect data on complications.^1,2,21,30^ Whilst 8 studies specified *a priori* specific events would be extracted as post-operative complications, these lists were not exhaustive and extracted an expanded list of complications in their results.^4,20,23,24,26–29^ Of the remaining five studies, methodology was non-specific as to what post-operative complication were to include, stating simply that post-operative complication data would be captured.^17–19,22,25^ Whilst prior specification of post-operative complications was heterogenous across the studies, data extraction of complications appears to have been generally well documented including medical and surgical complications. Overall the findings from this review demonstrate no increased risk of complications following outpatient shoulder arthroplasty as compared to arthroplasty performed in the inpatient setting. While the result of our meta-analysis suggests a statistically significant result, we must be aware of the selection bias present in the included studies, whereby we found that patients in these studies who underwent outpatient arthroplasty tended to be younger, male, have a lower BMI, and have less comorbidities. This highlights potential bias in outpatient shoulder arthroplasty studies due to comparison of older and more comorbid patients which are excluded in outpatient arthroplasty, but included for those undergoing inpatient arthroplasty. To account for these patient characteristic variables, a more appropriate analysis model would be a meta-regression analysis; however, studies lacked sufficient detail for this to be performed. This theory is supported by Erickson et al. who suggested that the reported lower complication rates seen in outpatient groups likely relate to their lower age and BMI when compared to those in the inpatient cohort.^19^ This highlights the need for a RCT to control for selection bias. Nevertheless, we must acknowledge that this bias is a real world necessity, as appropriate selection of patients is essential in order to minimise post-operative hospital readmission that have a significant effect on increasing costs and hospital LOS.^31^

While our findings of a bias towards selecting younger, healthier patients for outpatient shoulder arthroplasty is justifiable as a measure to reduce post-operative medical complications, an interesting finding is that studies incorporated in our review tended to select men more than women for outpatient arthroplasty. Inadequate reporting of data means that we are unable to comment on whether there was a difference in baseline characteristics of those presenting for surgery. Nevertheless, an important consideration is potential subconscious bias for referring men rather than women for outpatient procedure. Studies have shown that women presenting with pain to ED wait longer than men before receiving analgesia.^32,33^ It is possible that there exists a bias in physician perception of pain tolerance between genders. This could potentially result in a gender discrepancy in access to health services. Other literature suggests that physicians bias may already account for downplaying of symptoms of pain presented by women.^34^ Another argument would be that a patient’s sex may determine their willingness for outpatient procedure. Karlson et al.’s study suggested that women were more fearful of knee or hip arthroplasty and more likely to delay surgery with the hope of improved technology or until symptoms became much worse.^35^ It is possible that when presented with a fast-track discharge, men more than women were more willing to accept outpatient surgery. Awareness of these sex-based differences are important, as this exploring patient concerns when consenting for procedure may help to increase uptake of those suitable for outpatient arthroplasty. Further study for the reason of these sex-based differences is needed, with outpatient and inpatient groups matched for baseline patient characteristics.

Ode et al. suggest that outpatient procedures have the potential to cost 40.6% less than inpatient procedures.^2^ The major reason for the cost benefit of outpatient procedures is that patients do not require prolonged admission and occupy valuable bed space. Nonetheless, the few studies in our review that reported on costs of shoulder arthroplasty had large variability in their estimated saving. Some of this variability may be accounted for by choice of implant prosthesis, which are one of the modifiable factors related to operating room costs. Chalmers et al. suggest that operating room cost to contribute 70% of the overall costs related to shoulder arthroplasty. Non-modifiable factors such as patient level of comorbidity may also account for variations between the studies, as patient demographics and underlying health may affect how much cost benefit can be gained from outpatient procedures.^36^

However, cost alone should not be the driver behind moving more procedures to the outpatient setting. Whilst both financial and resource savings are important, the public perception and satisfaction of outpatient procedures will be damaged significantly if the earlier discharge results in a higher rate of representation to the emergency department. Nwankwo et al. reported 16.1% of outpatients versus 28.1% of inpatient had a post-operative ED visit, with Kramer finding 12.3% of outpatient versus 12.4% of inpatient had ED visits. Whilst optimisation of patients is important in order to reduce risks of representation and subsequent readmissions, it is also important to consider optimising the setting of outpatient procedures. Schairer et al. have shown that centres performing higher volumes of procedures have lower rates of complication and subsequent readmissions.^37^ The association of lower rates of complications at higher volume institutes is not unique to orthopaedic surgery.^25,38^ However, higher volume on its own should not be considered as having a direct association with outcome. Factors in determining lower complications and readmissions between higher and lower volume centres are complex and multifaceted. Nguyen et al. suggests two primary differences are the variation between local population health characteristics and the difference in the implementation of perioperative care pathways.^39^ They suggest that lower volume hospitals tend to receive sicker patients with greater comorbidity. Unlike high volume centres which rely on maintaining a systematic and standardised approach to perioperative care to manage high volumes, they suggest low volume centres are less strict in this. In lower volume centres, it is arguable that a systematic approach is far more crucial, as health professionals have less experience and require structure initially until this is developed. This suggests that centres seeking to implement outpatient arthroplasty should learn from high volume centres and implement a structured approach to perioperative care. Furthermore, those health professionals working in outpatient centres should be more experienced members of the surgical team, as there is less room for error given fast-track discharging of patients.

It should be noted that heterogeneity in analyses for readmissions and post-operative complications was substantial. In Glasziou and Sanders’s investigation into causes of heterogeneity, they identified several factors that may contribute to either artefactual or real variations in treatment effects.^40^ We identified three real factors that may have contributed significantly to the high heterogeneity seen in our analyses. Patient related factors, such as variation in comorbid status between participants; outcome related factors, such as variations in study size and length of follow-up between individual studies; and co-intervention related, such as the variations in use of nerve blocks and post-operative pain management plans between different studies. The high levels of heterogeneity mean we should proceed cautiously, as this acts to limit the weight that can be attributed to these findings.

A limiting factor to discharge can be suboptimal post-operative pain management. Whilst over half of the studies in our review commented on use of nerve blocks in management of patients, this equated to <10% of participants being known to have received a nerve block. Our experience is that nerve blocks are often more commonplace, and that the numbers reported may be an under representation due to inadequate reporting in database studies.^41^ A major factor in post-operative admission are complications of general anaesthesia. It is often common to focus on the increased risk of morbidity and mortality when choosing general over regional anaesthesia.^42^ However, post-operative issues such as urinary retention, pain and nausea and vomiting can increase patient LOS and add greater risk of hospital acquired infections and complications. Furthermore, the use of nerve blocks reduces the need for the prescription of opioids in the initial post-operative period. High opioids use can have a negative effect in the early post-operative period as it can further slow bowel motility, increase nausea and thereby lengthen the time until first oral intake, all increasing hospital LOS.^43^ The use of nerve blocks has a double effect as it both allow anaesthetists to maintain a lower level of general anaesthesia and can reduce early post-operative analgesic prescribing both reducing early post-operative complications. Morrison et al. demonstrated that poorly controlled post-operative pain had a statistically significant effect on increasing LOS, time to ambulation and resulted in worse outcomes long-term.^44^ The use of nerve blocks could potentially eliminate the need for admission due to uncontrolled pain following procedures, and improve rehabilitation in the early post-operative period, which could ultimately result in better patient reported outcomes.

Mears et al.’s evaluated factors contributing to increased length of hospital admission and identified four common factors associated with increased LOS following arthroplasty: female sex, older age, perioperative blood loss and ASA grade ≥3.^45^ Yet, whilst systems such as the ASA are universally recognised for assessing patients’ pre-operatively, purpose-built risk stratification tools such as the Outpatient Arthroplasty Risk Assessment score have demonstrated greater positive predictive values for favourable outcomes.^46^ Well-developed patient selection protocols or selection algorithms such as that of Fournier et al. are needed in order to help achieve timely discharges and prevent unnecessary admissions following outpatient procedures, which can be costly to the healthcare provider.^20^

### Limitations

Despite lower level and quality of evidence, this review identifies outpatient shoulder arthroplasty procedures are not associated with increased risk of complications, suggesting there is no sacrifice to patient safety, whilst reducing hospital LOS and cost associated with procedures. However, many database studies demonstrated a significant degree of selection bias in patient selection. The availability of solely observational data results in significant risk of bias in participant selection between comparator groups. Few studies reported on the difference in clinical outcomes between inpatient and outpatient procedures, with only two studies discussing presentation to emergency department. Protocols for post-operative pain management were limited. More consistency in reporting is needed to improve hospital LOS and ED presentations.

## Conclusion

Shoulder arthroplasty in the outpatient setting has a comparable safety profile to inpatient surgery with significant cost benefit. There is no demonstratable statistically significant difference with regards to readmissions between outpatient and inpatient shoulder arthroplasty. However, it is important to note that these findings are from low quality data and further high-quality literature is needed to confirm this. In the appropriately selected patient, outpatient shoulder arthroplasty is potentially safe and cost-effective; however, further research is needed and we should work towards understanding who the appropriate patients are for this post-operative care pathway.
